# Supplementary material for: STK11 is required for the normal program of ciliated cell differentiation in airways
Source: Cell Discov. 2019 Jul 16;5:36. doi: 10.1038/s41421-019-0104-z (PMC6796922; doi:10.1038/s41421-019-0104-z)
Supplement: Supplementary file 1 — Supplementary Information [file 41421_2019_104_MOESM1_ESM.pdf]

The Supplementary information file includes:

Supplementary Figs. S1 to S7.

Supplementary Table S1.

The list of primers used for qPCR_related to Material and Methods.

Supplementary Dataset S1.

The information of RNA-seq datasets_related to Fig 3.

Figure S1

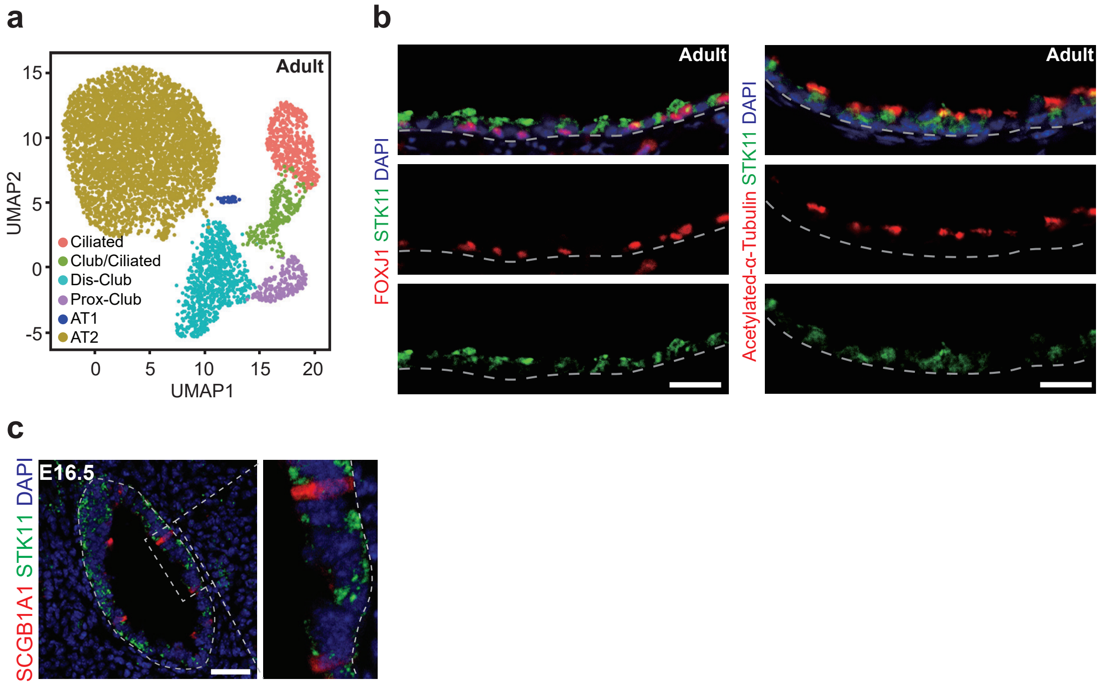
a
Adult
UMAP2
UMAP1
15
10
5
0
-5
0
5
10
15
20
Ciliated
Club/Ciliated
Dis-Club
Prox-Club
AT1
AT2
b
Adult
Adult
FOXJ1 STK11 DAPI
Acetylated-α-Tubulin STK11 DAPI
c
E16.5
SCGB1A1 STK11 DAPI

Figure S1. Analyzing of *Stk11* during ciliated cell development by scRNA-seq and immunostaining. **a** The UMAP plots of scRNA-seq of epithelial cells from adult lungs showed six clusters. **b** Immunofluorescence staining with antibodies against FOXJ1 (left), Acetylated-a-tubulin (right), and STK11 in lungs of adult mice. **c** Immunofluorescence staining with antibodies against STK11 and SCGB1A1 in E16.5 lungs. Scale bars: **b**: 20 μm; **c**: 25 μm.

Figure S2

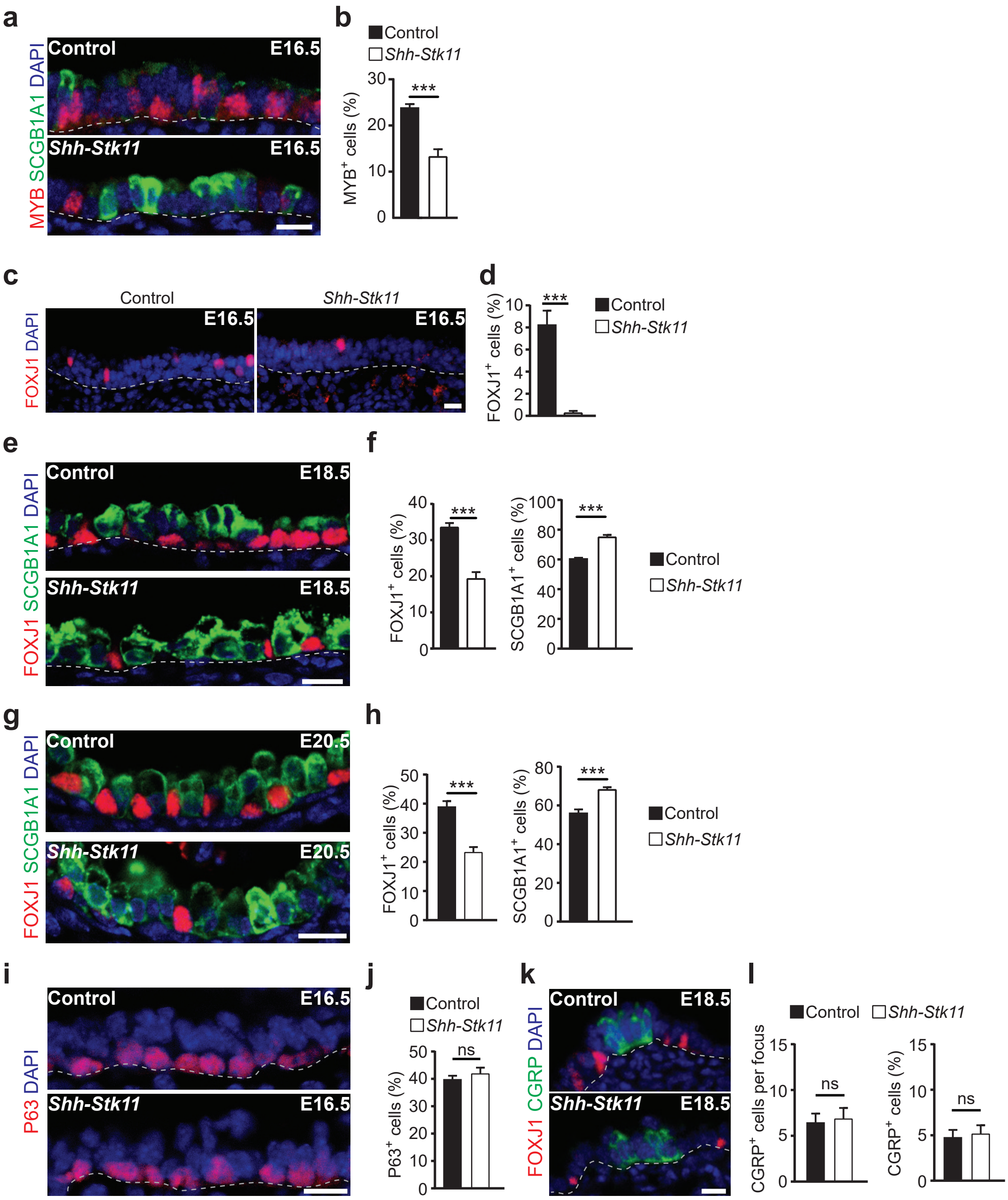

a
MYB SCGB1A1 DAPI
Control
E16.5
Shh-Stk11
E16.5
b
Control
Shh-Stk11
MYB+ cells (%)
***
c
FOXJ1 DAPI
Control
Shh-Stk11
E16.5
d
FOXJ1+ cells (%)
***
e
FOXJ1 SCGB1A1 DAPI
E18.5
f
FOXJ1+ cells (%)
SCGB1A1+ cells (%)
g
E20.5
h
i
P63 DAPI
j
P63+ cells (%)
ns
k
FOXJ1 CGRP DAPI
l
CGRP+ cells per focus
CGRP+ cells (%)

Figure S2. Loss of STK11 in airway progenitor cells impairs airway epithelial cell differentiation. **a** Immunofluorescence staining with antibodies against MYB and SCGB1A1 in E16.5 lungs. **b** The proportion of MYB^+ cells in the intrapulmonary airways of E16.5 lungs (Control, n=3; *Shh-Stk11*$^{F/F}$, n=4). **c** Immunofluorescence staining with an antibody against FOXJ1 in the trachea of E16.5 lungs. **d** The proportion of $FOXJ1^+$ ciliated cells in the tracheal epithelium of E16.5 lungs (n=4). **e** Immunofluorescence staining with antibodies against FOXJ1 and SCGB1A1 in E18.5 control lungs and *Shh-Stk11*$^{F/F}$ lungs. **f** The proportion of $FOXJ1^+$ cells in E18.5 lungs and the proportion of $SCGB1A1^+$ cells in E18.5 lungs (n=3). **g** Immunofluorescence staining with antibodies against FOXJ1 and SCGB1A1 in E20.5 control lungs and *Shh-Stk11*$^{F/F}$ lungs. **h** The proportion of $FOXJ1^+$ cells in E20.5 lungs and the proportion of $SCGB1A1^+$ cells in E20.5 lungs (Control, n=5; *Shh-Stk11*$^{F/F}$, n=8). **i** Immunofluorescence staining with an antibody against P63 in E16.5 lungs. **j** The proportion of $P63^+$ cells in the tracheal epithelium of E16.5 lungs (n=3). **k** Immunofluorescence staining with antibodies against FOXJ1 and CGRP in E18.5 lungs. **l** The $CGRP^+$ cell per focus and the proportion of $CGRP^+$ cells in E18.5 lungs (n=3). ns, not significant; ***, P<0.001. Data shown in the graphs are means ± SEM. Student's *t*-test. Scale bars: 11 μm.

Figure S3

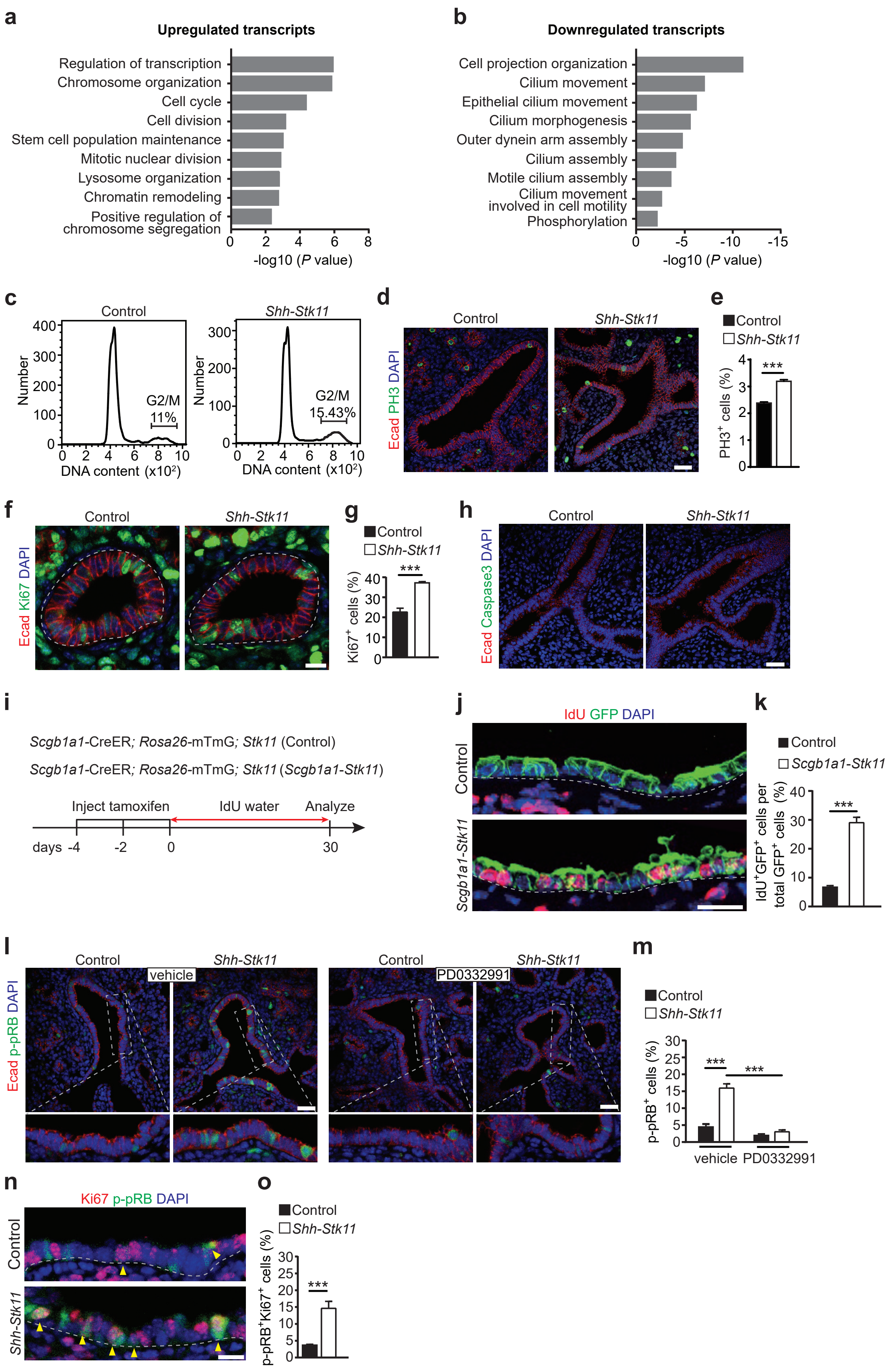

a
Upregulated transcripts
Regulation of transcription
Chromosome organization
Cell cycle
Cell division
Stem cell population maintenance
Mitotic nuclear division
Lysosome organization
Chromatin remodeling
Positive regulation of chromosome segregation
-log10 (P value)
b
Downregulated transcripts
Cell projection organization
Cilium movement
Epithelial cilium movement
Cilium morphogenesis
Outer dynein arm assembly
Cilium assembly
Motile cilium assembly
Cilium movement involved in cell motility
Phosphorylation
-log10 (P value)
c
Control
Shh-Stk11
Number
G2/M 11%
G2/M 15.43%
DNA content (x10²)
d
Ecad PH3 DAPI
e
Control
Shh-Stk11
PH3⁺ cells (%)
f
Ecad Ki67 DAPI
g
Ki67⁺ cells (%)
h
Ecad Caspase3 DAPI
i
Scgb1a1-CreER; Rosa26-mTmG; Stk11 (Control)
Scgb1a1-CreER; Rosa26-mTmG; Stk11 (Scgb1a1-Stk11)
Inject tamoxifen
IdU water
Analyze
days -4 -2 0 30
j
IdU GFP DAPI
Control
Scgb1a1-Stk11
k
IdU⁺GFP⁺ cells per total GFP⁺ cells (%)
l
vehicle
PD0332991
Ecad p-pRB DAPI
m
p-pRB⁺ cells (%)
vehicle PD0332991
n
Ki67 p-pRB DAPI
o
p-pRB⁺Ki67⁺ cells (%)

Figure S3. Increased cell proliferation in *Shh-Stk11*$^{F/F}$ lungs. **a-b** DAVID Gene Ontology (GO) analysis of genes that are up-regulated (a) or down-regulated (b) in the E16.5 *Shh-Stk11*$^{F/F}$ lungs as compared to Control lungs. **c** The cell-cycle progression status of epithelial cells in E16.5 lungs was analyzed with PI staining. **d** E16.5 lungs were stained with an antibody against Phospho-Histone H3 (PH3). **e** The proportion of PH3^{+} cells in the intrapulmonary airways of E16.5 lungs (n=3). **f** Immunofluorescence staining with antibodies against Ki67 and Ecad in E16.5 lungs. **g** The proportion of Ki67^{+} intrapulmonary airway epithelial cells in E16.5 lungs (n=4). **h** Immunofluorescence staining with antibodies against Caspase3 and Ecad in E16.5 lungs. **i** 10 week-old *Scgb1a1*-CreER; *Rosa26*-mTmG; *Stk11*$^{F/+}$ (Control) and *Scgb1a1*-CreER; *Rosa26*-mTmG; *Stk11*$^{F/F}$ (*Scgb1a1-Stk11*$^{F/F}$) mice were treated with three doses of tamoxifen and given IdU water for 30 days before analyzing. **j** Immunofluorescence staining with antibodies against IdU and GFP at day 30. **k** The proportion of IdU^{+} cells in the GFP labeled intrapulmonary airway epithelium after IdU water treatment for 30 days (n=4). **l** Immunofluorescence staining with antibodies against Ecad and p-pRB in E16.5 lungs after the vehicle or PD0332991 treatment. **m** The proportion of p-pRB^{+} cells in the intrapulmonary airway epithelium after PD0332991 treatment (vehicle Control, n=3; vehicle *Shh-Stk11*$^{F/F}$, n=3; PD0332991 Control, n=4; PD0332991 *Shh-Stk11*$^{F/F}$, n=3). **n** Immunofluorescence staining with antibodies against Ki67 and p-pRB in E16.5 lungs. **o** The proportion of Ki67^{+}p-RB^{+} cells in the Ki67^{+} intrapulmonary airway epithelial cells in E16.5 lungs (n=4). ***, *P*<0.001. Data shown in the graphs are means ± SEM. Student's *t*-test. Scale bars: **d**, **h**, **l**: 25 μm; **f**, **n**: 11 μm. **j**: 20 μm.

Figure S4

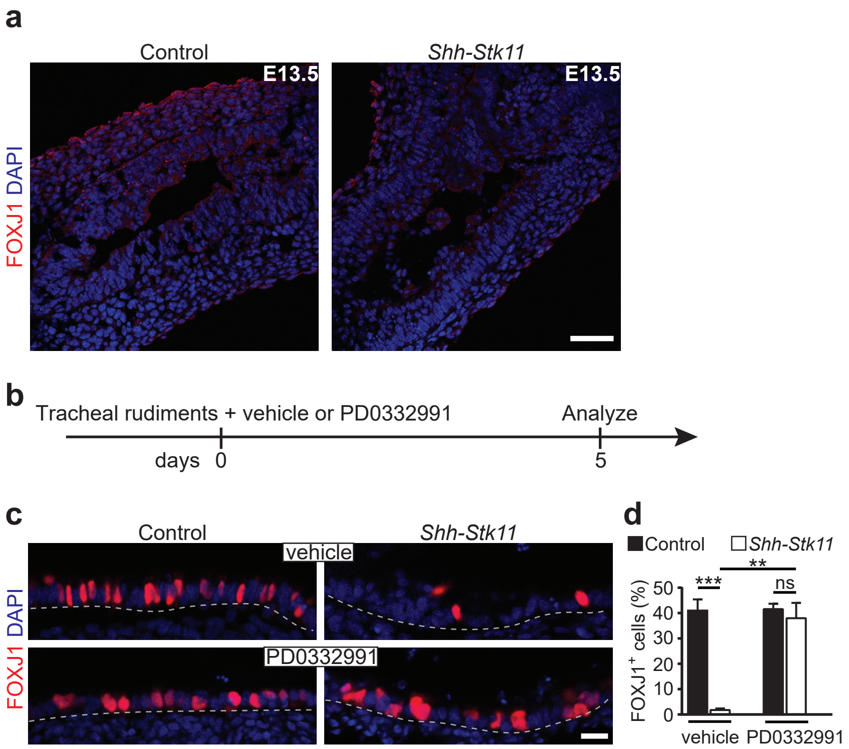
a
Control
Shh-Stk11
E13.5
E13.5
FOXJ1 DAPI
b
Tracheal rudiments + vehicle or PD0332991
Analyze
days 0
5
c
Control
Shh-Stk11
vehicle
PD0332991
FOXJ1 DAPI
d
Control
Shh-Stk11
**
***
ns
FOXJ1+ cells (%)
50
40
30
20
10
0
vehicle
PD0332991

Figure S4. Inhibition of cell proliferation can rescue ciliated cell differentiation defect in the *Stk11*-deficient tracheal rudiments. **a** Immunofluorescence staining with an antibody against FOXJ1 in the tracheal rudiments of E13.5 lungs. **b** E13.5 tracheal rudiments were cultured with vehicle or PD0332991 for 5 days. **c** Immunofluorescence staining with an antibody against FOXJ1 in the tracheal rudiments at day 5. **d** The proportion of $FOXJ1^+$ ciliated cells in cultured tracheal rudiments at day 5 (n=3). ns, not significant; **, $P<0.01$; ***, $P<0.001$. Data shown in the graphs are means ± SEM. Student's *t*-test. Scale bar: **a**: 25 μm; **c**: 11 μm.

Figure S5

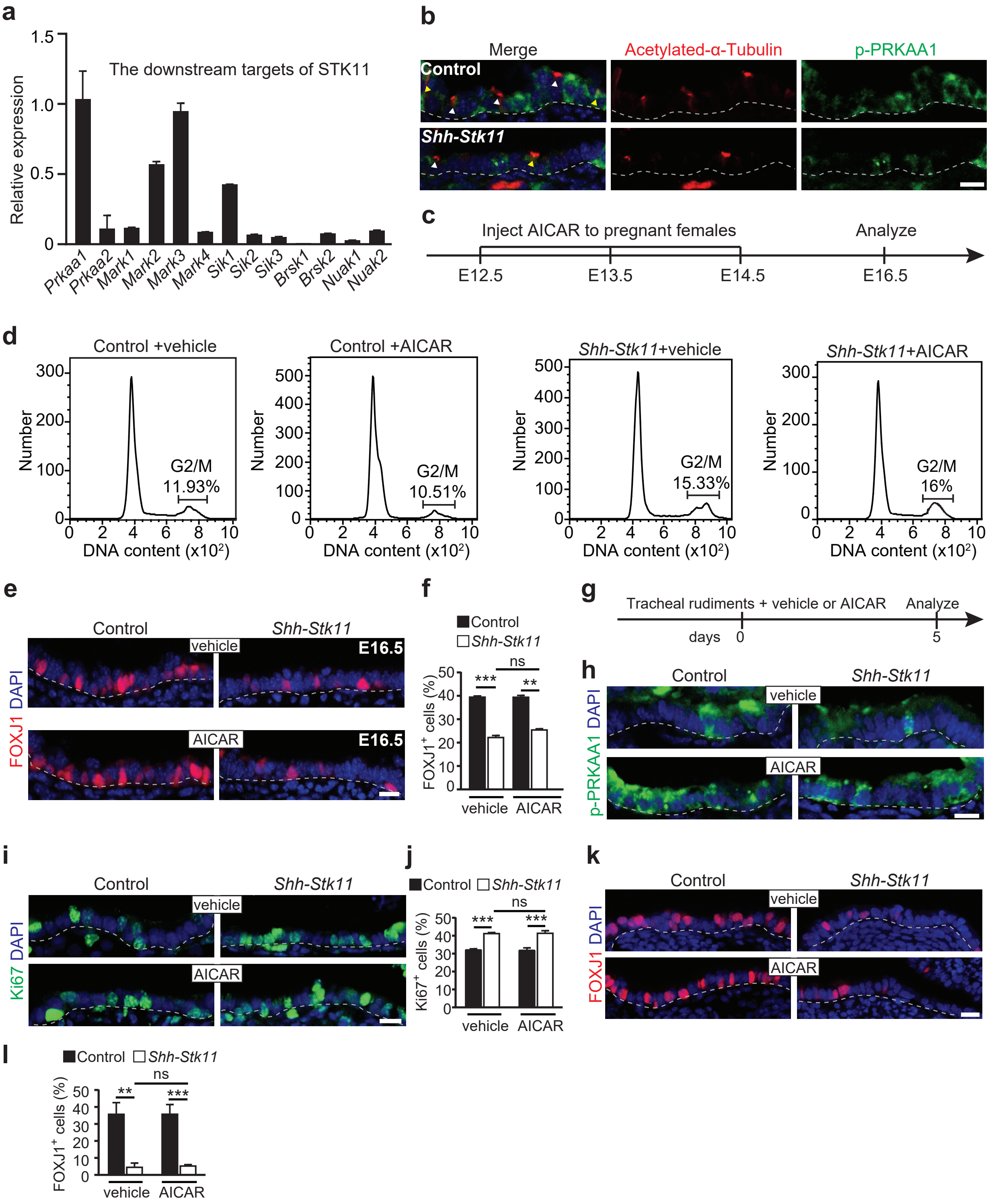

a
The downstream targets of STK11
Relative expression
Prkaa1 Prkaa2 Mark1 Mark2 Mark3 Mark4 Sik1 Sik2 Sik3 Brsk1 Brsk2 Nuak1 Nuak2
b
Merge
Acetylated-α-Tubulin
p-PRKAA1
Control
Shh-Stk11
c
Inject AICAR to pregnant females
Analyze
E12.5
E13.5
E14.5
E16.5
d
Control +vehicle
Control +AICAR
Shh-Stk11+vehicle
Shh-Stk11+AICAR
G2/M 11.93%
G2/M 10.51%
G2/M 15.33%
G2/M 16%
Number
DNA content (x10²)
e
Control
Shh-Stk11
vehicle
AICAR
E16.5
FOXJ1 DAPI
f
Control
Shh-Stk11
FOXJ1⁺ cells (%)
vehicle AICAR
ns
g
Tracheal rudiments + vehicle or AICAR
Analyze
days 0
5
h
Control
Shh-Stk11
vehicle
AICAR
p-PRKAA1 DAPI
i
Control
Shh-Stk11
vehicle
AICAR
Ki67 DAPI
j
Control Shh-Stk11
Ki67⁺ cells (%)
vehicle AICAR
ns
k
Control
Shh-Stk11
vehicle
AICAR
FOXJ1 DAPI
l
Control Shh-Stk11
FOXJ1⁺ cells (%)
vehicle AICAR
ns

Figure S5. Activating p-PRKAA1 cannot rescue impaired ciliated cell differentiation both *in vivo* and *in vitro*. **a** The mRNA levels of STK11-downstream genes in the epithelial cells of E16.5 lungs (n=3). **b** Immunofluorescence staining with antibodies against p-PRKAA1 and Acetylated-α-Tubulin in E16.5 lungs. White arrows indicate ciliated cells positive for Acetylated-α-Tubulin but negative for p-PRKAA1. Yellow arrows indicate ciliated cells positive for both Acetylated-α-Tubulin and p-PRKAA1. **c** Pregnant mice were treated with AICAR by intraperitoneal injection from E12.5 to E14.5 each day. Lungs were analyzed at E16.5. **d** The cell-cycle progression status of epithelial cells in E16.5 lungs was analyzed by PI staining. **e** Immunofluorescence staining with an antibody against FOXJ1 in the E16.5 lungs after the vehicle or AICAR treatment. **f** The proportion of FOXJ1$^+$ cells in the intrapulmonary airways of E16.5 lungs after the treatments (n=3). **g** E13.5 tracheal rudiments were cultured with AICAR for 5 days. **h** Cultured tracheal rudiments were stained with an antibody against p-PRKAA1 at day 5 after AICAR treatment. **i** Cultured tracheal rudiments were stained with antibodies against Ki67 at day 5. **j** The proportion of Ki67$^+$ epithelial cells in the cultured tracheal rudiments at day 5 (vehicle Control, n=3; vehicle *Shh-Stk11*$^{F/F}$, n=3; AICAR Control, n=3; AICAR *Shh-Stk11*$^{F/F}$, n=4). **k** Cultured tracheal rudiments were stained with an antibody against FOXJ1 at day 5. **l** The proportion of FOXJ1$^+$ ciliated cells in cultured tracheal rudiments at day 5 (vehicle Control, n=4; vehicle *Shh-Stk11*$^{F/F}$, n=4; AICAR Control, n=3; AICAR *Shh-Stk11*$^{F/F}$, n=3). ns, not significant; **, P<0.01; ***, P<0.001. Data shown in the graphs are means ± SEM. Student's *t*-test. Scale bars: 11 μm.

Figure S6

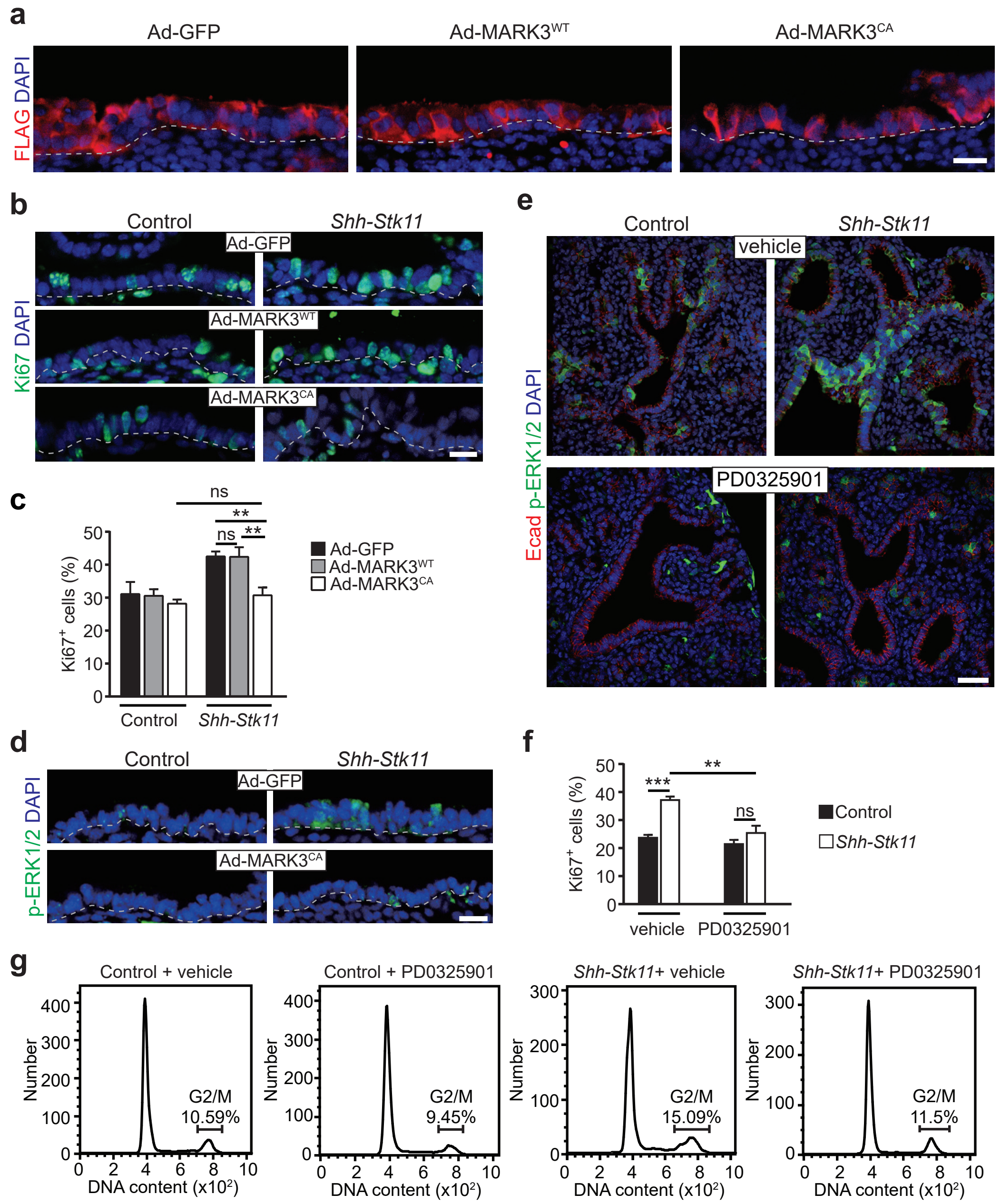

a
Ad-GFP
Ad-MARK3WT
Ad-MARK3CA
FLAG DAPI
b
Control
Shh-Stk11
Ad-GFP
Ad-MARK3WT
Ad-MARK3CA
Ki67 DAPI
c
ns
**
ns
**
Ki67+ cells (%)
50
40
30
20
10
0
Control
Shh-Stk11
Ad-GFP
Ad-MARK3WT
Ad-MARK3CA
d
Control
Shh-Stk11
Ad-GFP
Ad-MARK3CA
p-ERK1/2 DAPI
e
Control
Shh-Stk11
vehicle
PD0325901
Ecad p-ERK1/2 DAPI
f
***
**
ns
Ki67+ cells (%)
50
40
30
20
10
0
vehicle
PD0325901
Control
Shh-Stk11
g
Control + vehicle
Control + PD0325901
Shh-Stk11+ vehicle
Shh-Stk11+ PD0325901
Number
400
300
200
100
0
G2/M
10.59%
9.45%
15.09%
11.5%
0 2 4 6 8 10
DNA content (x10²)

Figure S6. STK11/MARK3 cascade controls ciliated cell differentiation by limiting the activity of ERK1/2 signaling. **a** Cultured tracheal rudiments were stained with an antibody against Flag at day 5 to indicate the adenovirus infection efficiency. **b** Cultured tracheal rudiments were stained with antibodies against Ki67 at day 5 after adenovirus infection. **c** The proportion of $Ki67^+$ epithelial cells in the cultured tracheal rudiments were quantified (n=3). **d** Cultured tracheal rudiments were stained with antibodies against p-ERK1/2 at day 5 after adenovirus infection. **e** Immunofluorescence staining with antibodies against p-ERK1/2 and Ecad in E16.5 lungs after the vehicle or PD0325901 treatment. **f** The proportion of $Ki67^+$ cells in the intrapulmonary airways of E16.5 lungs after PD0325901 treatment (vehicle Control, n=4; vehicle *Shh*-*Stk11*$^{F/F}$, n=4; PD0325901 Control, n=9; PD0325901 *Shh*-*Stk11*$^{F/F}$, n=7). **g** The cell-cycle status of epithelial cells in E16.5 lungs after the vehicle or PD0325901 treatment were analyzed with PI staining. ns, not significant; **, *P*<0.01; ***, *P*<0.001. Data shown in the graphs are means ± SEM. Student's *t*-test. Scale bars: **a**, **b**, **d**: 11 μm; **e**: 25 μm.

Figure S7

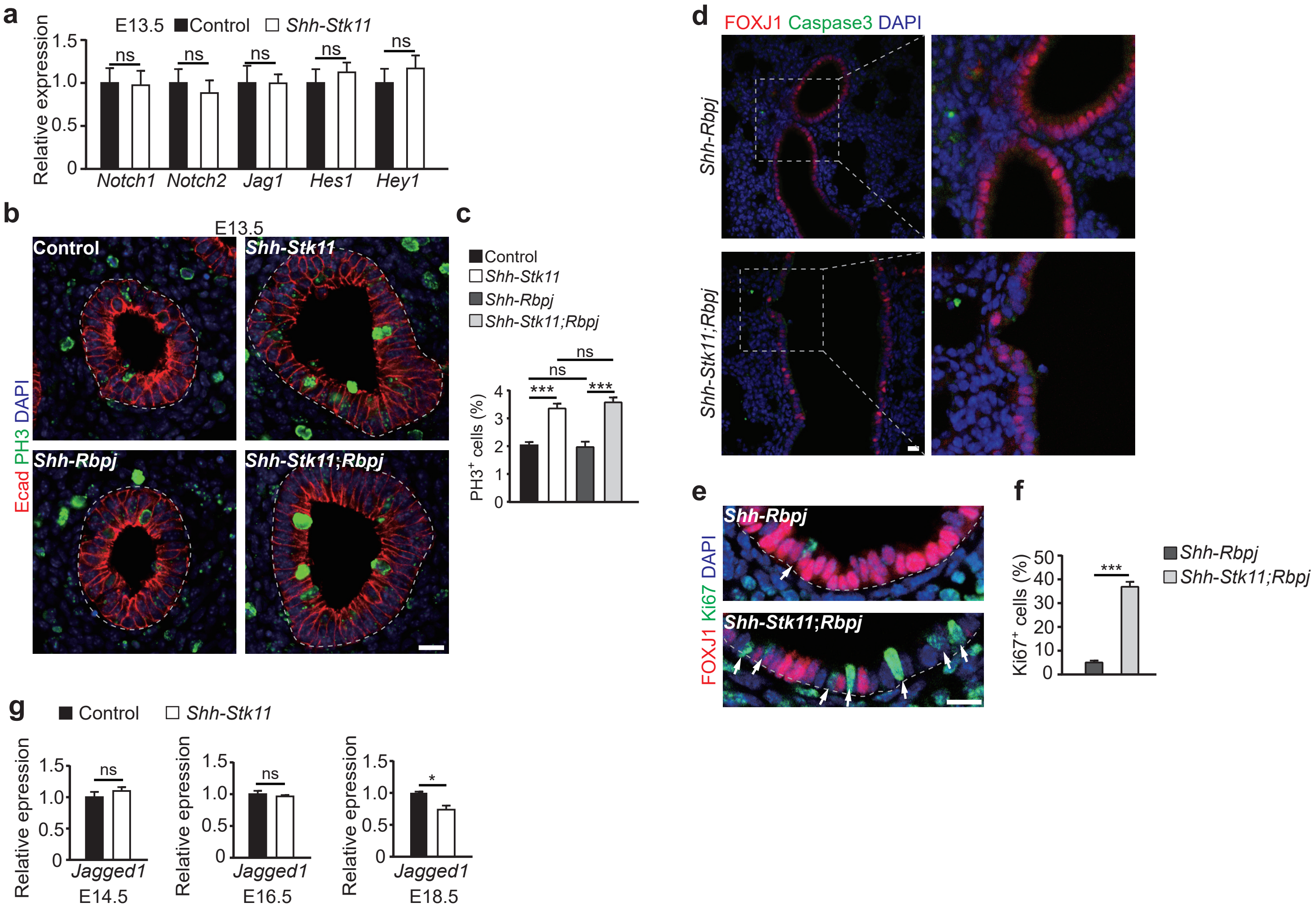

a
E13.5 Control Shh-Stk11
Relative expression
1.5
1.0
0.5
0
ns
Notch1 Notch2 Jag1 Hes1 Hey1
b
E13.5
Control
Shh-Stk11
Shh-Rbpj
Shh-Stk11;Rbpj
Ecad PH3 DAPI
c
Control
Shh-Stk11
Shh-Rbpj
Shh-Stk11;Rbpj
PH3+ cells (%)
ns
***
d
FOXJ1 Caspase3 DAPI
Shh-Rbpj
Shh-Stk11;Rbpj
e
Shh-Rbpj
Shh-Stk11;Rbpj
FOXJ1 Ki67 DAPI
f
Ki67+ cells (%)
50
40
30
20
10
0
***
Shh-Rbpj
Shh-Stk11;Rbpj
g
Control Shh-Stk11
Relative epression
ns
*
Jagged1
E14.5
E16.5
E18.5

Figure S7. The function of STK11 in regulating ciliated cell differentiation is independently of Notch signaling. **a** The mRNA expression level of Notch signaling pathway genes in the E13.5 Control lungs and *Shh-Stk11*$^{F/F}$ lungs. **b** Immunofluorescence staining with antibodies against PH3 and Ecad in E13.5 Control lungs, *Shh-Stk11*$^{F/F}$ lungs, *Shh-Rbpj*$^{F/F}$ lungs, and *Shh-Stk11*$^{F/F}$; *Rbpj*$^{F/F}$ lungs. **c** The proportion of PH3$^+$ airway epithelial cells in the intrapulmonary airways of E13.5 lungs (Control, n=4; *Shh-Stk11*$^{F/F}$, n=3; *Shh-Rbpj*$^{F/F}$, n=4; *Shh-Stk11*$^{F/F}$; *Rbpj*$^{F/F}$, n=3). **d** Immunofluorescence staining with antibodies against Caspase3 and FOXJ1 in E16.5 *Shh-Rbpj*$^{F/F}$ lungs and *Shh-Stk11*$^{F/F}$; *Rbpj*$^{F/F}$ lungs. **e** Immunofluorescence staining with antibodies against FOXJ1 and Ki67 in E16.5 lungs. White arrows indicate the FOXJ$^-$Ki67$^+$ cells. **f** The proportion of Ki67$^+$ cells in the intrapulmonary airways of E16.5 lungs (*Shh-Rbpj*$^{F/F}$, n=4; *Shh-Stk11*$^{F/F}$; *Rbpj*$^{F/F}$, n=6). **g** The mRNA level of *Jagged1* in the epithelia of Control and *Shh-Stk11*$^{F/F}$ lungs at E14.5, E16.5, and E18.5. ns, not significant, *, P<0.05; ***, P<0.001. Data shown in the graphs are means ± SEM. Student's *t*-test. Scale bars: **b**, **d**, **e**: 11 μm.

Table S1. Sequences of DNA primers used for RT-qPCR

Gene	Forward sequence (5'-3')	Reverse sequence (5'-3')
Gapdh	CATCACTGCCACCCAGAAGACTG	ATGCCAGTGAGCTTCCCGTTCAG
Myb	GAAAGTGCCTCACCAGCAAGGT	CGAGCTTTCATGGTTGCTGGAAG
Mcidas	GTGGAAGTCCTTTTCGGGATGC	TAGGAGACGCTTCGGTTCCGAG
Foxj1	CTCCTATGCCACTCTCATCTGC	GACAGGTTGTGGCGGATGGAAT
Scgb1a1	GGTTATGTGGCATCCCTGAAGC	GCTTACACAGAGGACTTGTTAGG
Muc5b	CTGAAGACCTGTCGGAACCCAA	GCCACACACTTCATCTGGTCCT
Ranbp2	ATGGTTGCTGGCGAAGTGCTGA	ATCTTCTGCCCATCGAGGTGGT
Smc5	TGCGTGATACGAACAGGCACCT	GCAAGTATCCTGTTCCATCAGCC
Mki67	GAGGAGAAACGCCAACCAAGAG	TTTGTCCTCGGTGGCGTTATCC
Sgo2a	ACCACAGGACACAGAAGTCGAC	GTTGAGAGGGACGCACTGTCTT
Taf1	ATCCTACAGGCTGTGGTGAAGG	TTCAGAGACAGGCGACGAAGGT
Ccnd2	GCAGAAGGACATCCAACCGTAC	ACTCCAGCCAAGAAACGGTCCA
Cenpe	AGGATCATGCCACCGAGAAGAC	GCTGTGTCTCTTGGAGTTTCTGG
Prkaa1	GGTGTACGGAAGGCAAAATGGC	CAGGATTCTTCCTTCGTACACGC
Prkaa2	CTGAAGCCAGAGAATGTGCTGC	GAGATGACCTCAGGTGCTGCAT
Mark1	TGAAGGTCCAGCGGAGCATCTC	CCTCTTGGTGTATGAGACGGCA
Mark2	GAACCTCTCCCTGACTACAAGG	GGAGCAGATAGGTAGCCATCAC
Mark3	CAGTCTCCTCATCACAAAGGGC	TGCTGGTCTGACTCCTCTTTGG
Mark4	CTGCGGAGATTTCTGGTGCTGA	TCCGTGTATGGCTTCAGCTCCT
Sik1	GCGACTACAACGAACAGGTGCT	GTAGGAGGTAGTAAATGGCGGC
Sik2	GCTGCCTTTATGGAGGAAGAGTG	GAGGTCTCCATCATACTGCTGG
Sik3	AACGGCAGCTAGGACAACAGTC	GTGTTGGAGTCCTTGTAGGTGG
Brsk1	GACGTTCTGGAAAGCATGGCGT	GGTCTTCACAGCTAGGATACCG
Brsk2	CGGAAAGAACGGTATCCAAGCC	CATCTGTCACACTGAGCACTTCC
Nuak1	CAGATCAGCAGCGGAGAGTACC	CAATGGTTGGCGATGTCCTCGA
Nuak2	CTGGTGAAGCAAATCAGTAACGG	CCACCAATGACTGGCTACATCC
Notch1	GCTGCCTCTTTGATGGCTTCGA	CACATTCGGCACTGTTACAGCC
Notch2	CCACCTGCAATGACTTCATCGG	TCGATGCAGGTGCCTCCATTCT
Jag1	TGCGTGGTCAATGGAGACTCCT	TCGCACCGATACCAGTTGTCTC
Hes1	GGAAATGACTGTGAAGCACCTCC	GAAGCGGGTCACCTCGTTCATG
Hey1	CCAACGACATCGTCCCAGGTTT	CTGCTTCTCAAAGGCACTGGGT
